# Enrichment of Phenolic Compounds from Olive Mill Wastewater and* In Vitro* Evaluation of Their Antimicrobial Activities

**DOI:** 10.1155/2017/3706915

**Published:** 2017-12-28

**Authors:** Saleh Abu-Lafi, Mahmoud Sami Al-Natsheh, Reem Yaghmoor, Fuad Al-Rimawi

**Affiliations:** ^1^Faculty of Pharmacy, Al-Quds University, P.O. Box 20002, Jerusalem, State of Palestine; ^2^Chemistry Department, Faculty of Science and Technology, Al-Quds University, P.O. Box 20002, Jerusalem, State of Palestine; ^3^Biology Department, Faculty of Science and Technology, Al-Quds University, P.O. Box 20002, Jerusalem, State of Palestine

## Abstract

The production of olive oil generates massive quantities of by-product called olive mill wastewater (OMWW). The uncontrolled disposal of OMWW poses serious environmental problems. The OMWW effluent is rich in several polyphenolic compounds. Liquid-liquid extraction of OMWW using ethyl acetate solvent was used to enrich phenolic compounds under investigation. Total phenolic and flavonoid content and antioxidant activity of the extract were determined. HPLC coupled to photodiode array (PDA) detector was used to analyze the main three phenolic compounds of OMWW, namely, hydroxytyrosol, tyrosol, and oleuropein. The antimicrobial activity of the extract was also investigated. Additionally, the OMWW extract was used as natural preservative and antioxidants for olive oil. Results showed that OMWW is very rich in phenolic compounds and has strong antioxidant activity. HPLC analysis showed that the extract contains mainly hydroxytyrosol and tyrosol but no oleuropein. The OMWW extract showed also positive activities as antibacterial (gram positive and gram negative) and antifungal as well as activities against yeast. The addition of OMWW extract to olive oil samples has an effect on the stability of olive oil as reflected by its acid value, peroxide value, *K*_232_ and *K*_270_, and total phenolic content.

## 1. Introduction

**Figure 1:**
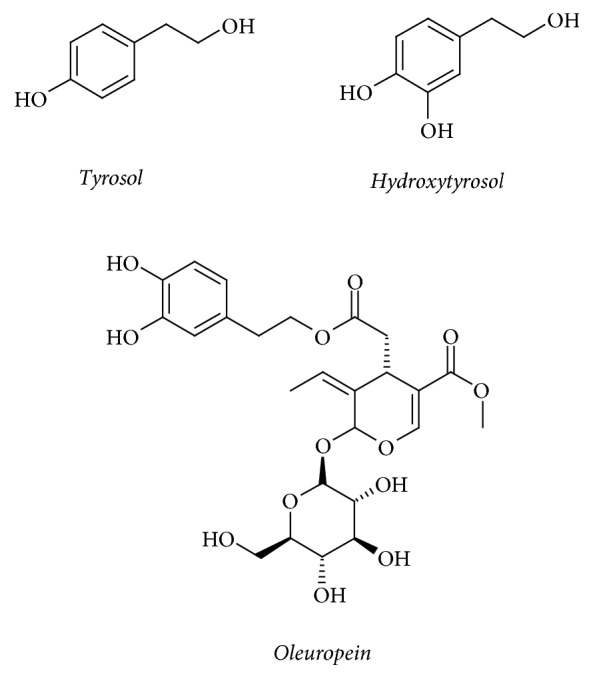
Structure of the main three phenolic compounds present in OMWW.

Olive oil production accumulates large amounts of olive mill wastewater (OMWW) remnants. The OMWW is known to be one of the most polluting effluents produced by the agrofood industries because of its high polluting phytotoxins [1]. It exhibits high toxicity towards plants, bacteria, and aquatic organisms, due to its composition of organic substances (14-15%) and phenolic compounds (up to 10 g/l) [1]. However, this perception is changing since OMWW has the potential to become a low-cost starting material rich in bioactive compounds, particularly phenolics which can be extracted, enriched, and ultimately applied as natural antioxidants in food, cosmetics, and pharmaceutical industries [2, 3]. In many developing countries, most olive millers either use common treatment methods like evaporation to manage OMWW or dispose it in areas surrounding their facilities [1]. OMWW contains a lot of phenolic compounds of which three phenolic compounds, tyrosol, hydroxytyrosol, and oleuropein (Figure 1), are the main components.

The antioxidant activity of these phenolics has been attributed to different mechanisms, among which are prevention of chain initiation, decomposition of peroxides, prevention of continued hydrogen abstraction, binding of transition metal ion catalysts, reductive capacity, and radical scavenging [4]. Extraction of tyrosol and other phenolic antioxidants from OMWW was investigated [1, 5, 6].

In Palestine, OMWW total phenolics, flavonoid content, antioxidant activities, or OMWW role as a potential preservative in olive oil has never been investigated. The objectives of this work is to extract polyphenolic compounds from OMWW, to analyze them by using HPLC-PDA, and to evaluate antimicrobial activity of OMWW and to use it as natural antioxidant and preservative for olive oil.

## 2. Materials and Methods

### 2.1. OMWW Collection

Twenty liters of OMWW was collected from an olive mill in a village located in Hebron at the southern part of Palestine.

### 2.2. Pretreatment of the Sample

The sample was filtered from solid particles using filter paper and then centrifuged at 402 ×g for 15 minutes.

### 2.3. Treatment of Sample

n-Hexane was added to the filtered sample (1 : 1, v/v) and left for one hour and then centrifuged at 4000 rpm for 15 minutes to remove all the fats and oils from the sample. The n-hexane layer was discarded.

### 2.4. Extraction of Phenolic Compounds from Treated Sample

The extraction procedure was performed using ethyl acetate (1 : 2, v/v, OMWW/ethyl acetate). The sample is then transferred to a beaker with large headspace and stirred at 120 rpm for 30 minutes. The upper layer which contains the phenolic compounds was separated. Finally, ethyl acetate was evaporated under vacuum using rotary evaporator at 40°C until crude viscous extract was obtained.

### 2.5. Determination of Total Phenolic Content

Total phenolic contents were determined using Folin-Ciocalteu phenol reagent [7], where the extract (40 *μ*l) was mixed with 1.8 ml of Folin-Ciocalteu reagent (which was prediluted 10-fold using distilled water) and allowed to stand for 5 minutes at room temperature, and then 1.2 ml of sodium bicarbonate (with a concentration of 7.5%) was added to the mixture. After sixty minutes at room temperature, absorbance was measured at 765 nm. Aqueous solutions of gallic acid (concentrations in the range of 100−500 ppm) were used for calibration. Results were expressed as gram of gallic acid equivalents (GAE) per liter of OMWW sample.

### 2.6. Total Flavonoids Content (TFC)

Total flavonoids content was performed according to the method of Kim et al. 2003 [8]. Distilled water (4 ml) was added to 1 ml of OMWW extract, then 0.3 ml of 5% sodium nitrite solution was added, followed by 0.3 ml of 10% aluminum chloride solution. Test tubes were incubated at ambient temperature (25°C) for 5 minutes, and then 2 ml of 1 M sodium hydroxide was added to the mixture. The volume of reaction mixture was made to 10 ml with distilled water. The mixture was vortexed and the absorbance of the pink color developed was determined at 510 nm. Aqueous solutions of catechin (with concentrations of 50–100 ppm) were used for calibration, and the results were expressed as mg catechin equivalents (CEQ) per liter of OMWW sample [9].

### 2.7. Antioxidant Activity Determined by FRAP

Antioxidant activity using FRAP method of the extracts was determined using the method of ferric reducing/antioxidant power (FRAP) of Benzie and Strain, 1999 [10]. First, 3 mL of FRAP reagent was warmed at 37°C and added to 40 *μ*l of each extract and the mixtures were incubated at 37°C for 4 minutes. Then, absorbance was read at 593 nm with reference to blank containing distilled water incubated at 37°C for one hour. Aqueous solutions of Fe^+2^ with concentrations of 2–5 mM were used for calibration. Results were expressed as mM Fe^+2^ per liter of OMWW sample.

### 2.8. Antioxidant Activity Determined by DPPH

Antioxidant activity of the extracts was determined by DPPH free radical method. The determination of the DPPH radical scavenging activity was performed according to method of Brand-Williams et al. [11]. Samples were reacted with DPPH radical in ethanol solution, where 0.5 mL of the extracts, 3 mL of absolute ethanol, and 0.3 mL of DPPH solution (0.5 mM in ethanol) were mixed. Absorbance was measured at 517 nm after 100 min of reaction using UV/VIS spectrophotometer. A mixture of ethanol (3.3 mL) and sample (0.5 mL) serve as blank, while the control solution was prepared by mixing 3.5 mL ethanol and 0.3 mL DPPH radical solution. Results were expressed as mg Trolox per liter of OMWW sample.

### 2.9. Antioxidant Activity Determined by ABTS

ABTS (2,2-azino-di-(3-ethyl-ethylbenzothiazoline-sulphonic acid)) method was performed according to Re et al., 1999 [12]. 7 mM of ABTS^+^ stock solution was prepared by reaction of 7 mM ABTS solution and 2.45 mM of potassium persulphate (K_2_S_2_O_8_). The working solution of ABTS^+^ was obtained by diluting the stock solution in ethanol to get an absorbance of 0.70 ± 0.02 at wavelength of 734 nm. 50 *μ*L of sample extract was added to 90 *μ*L of ABTS^+^ solution and the absorbance was taken at 734 nm at 30°C after 10 minutes of initial mixing. The radical scavenging activity was expressed as Trolox equivalent antioxidant capacity (TEAC): mg Trolox per liter OMWW.

### 2.10. Antioxidant Activity Determined by CUPRAC

Cupric reducing antioxidant capacity (CUPRAC) method was performed according to Apak et al. [13]. The reaction mixture consisted of 0.5 ml of extract, 1 ml of copper(II) chloride solution (0.01 M prepared from CuCl_2_·2H_2_O), 1 ml of ammonium acetate buffer at pH 7.0, and 1 ml of neocuproine solution (0.0075 M). The volume of the mixture was made 4.1 ml by adding 0.6 ml of distilled water, and then total mixture was incubated for 1 hour at room temperature. The absorbance of the solutions were measured at 450 nm. Results were expressed as mg Trolox per liter of OMWW sample.

### 2.11. HPLC-PDA Analysis

HPLC (Waters Alliance e2695 module) equipped with photodiode array (2998 PDA) was used to determine hydroxytyrosol, tyrosol, and oleuropein in the crude extracts of OMWW. Data acquisition and control were carried out using Empower 3 chromatography data software (Waters, Germany). Octadecyl (ODS) column (XBridge, 4.6 ID × 150 mm, 5 *μ*m, Waters, Germany) with a guard column of XBridge ODS (20 mm × 4.6 mm ID, 5 *μ*m, Waters, Germany) was used. The mobile phase consisted of a gradient mixture of 0.5% acetic acid solution (A) and acetonitrile (B). A 100% of solvent A was used and then increased to 70% A in 40 minutes, then to 40% A in 20 minutes, then to 10% A in 2 minutes and stayed for 6 minutes and then back to the starting percentages in 2 minutes. The equilibration time was 5 minutes with 100% A. A 0.45 *μ*m PTFE filter was used for all the samples prior to injection to the HPLC. Scanning wavelengths range of 210–500 nm was used. The flow rate was 1 ml/min and the injection volume was 10 *μ*l. Column temperature was at ambient temperature.

The PDA detector was utilized to use the maximum wavelength of each separated polyphenol. Calibration curve that covers linear interval was constructed to quantify all the polyphenols under investigation. The common suitable wavelength was at 279 nm.

### 2.12. Antibacterial and Anticandidal Activity by Well Diffusion Method

Antibacterial activities of the extracts were investigated by well diffusion method which depends on diffusion of the sample tested from a vertical cylinder through a solidified agar layer in a plate. In this method, the media were prepared by mixing 3.05 grams of Muller Hinton agar in 100 mL of distilled water for each microorganism.* Candida albicans* yeast with both gram positive* (Staphylococcus aureus)* and gram negative* (Escherichia coli)* bacteria was tested. The medium was boiled and then sterilized at 121°C for 15 minutes. After sterilization, the medium was cooled, then at 45°C it was poured in sterile Petri dishes (20 mL/plate). After solidification of the media, four holes were made using sterile cylinder with a diameter of 6 ± 0.1 mm. Bacterial and candidal suspensions were prepared by inoculation of the selected specimens in Muller Hinton broth, incubated at 37°C for 24 hours, and then adjusted to have 10^8^ cfu/ml using spectrophotometer at 625 nm (optical density 0.08–0.1). An inoculum of 10^6^ cfu/ml of bacterial suspension was prepared by diluting 0.1 ml of the prepared bacterial broth culture with 9.9 ml of sterile saline. Candida specimen was used undiluted (10^8^ cfu/ml). Each bacterial suspension was distributed on the surface of Muller Hinton agar plate using a cotton applicator. 100 *μ*L of each extract was placed in each hole for each plate. 100 *μ*L of the solvent containing the extract was poured in the third hole as negative control, and standard antibiotics were used for each bacterial specimen as positive control. The plates were incubated at 37 ± 0.5°C for 24 hours. After incubation period, the zone of inhibition was measured by a caliper.

### 2.13. Antifungal Activity

Muller Hinton agar was prepared in tubes; the tubes were melted by heating in water bath. After that the OMWW extract (100 *μ*l) is added to one of the tubes at 45°C, while the other tube is let without extract (to be used as positive control). The mixture is poured in a Petri dish and let to cool. A portion of an* Aspergillus niger *colony with a diameter of 0.5 mm was put on the center of the plate surface. The plate was incubated at 37°C for one week, and then the diameter of the colony was measured and compared to that on positive control (agar without OMWW extract) that was prepared following the same steps.

### 2.14. Effect of Addition of OMWW Extract on Quality of Olive Oil

OMWW extract was added to olive oil sample of different concentrations, and the effect on the oil quality was assessed by measuring different oil parameters (acid value, peroxide value, *K*_232_ and *K*_270_) in the day of preparation (time: zero) and after three months of storage at room temperature in dark and dry place. The oil samples were put in amber glass bottles.

#### 2.14.1. Acid Value

Acid value was determined by using the AOAC method number 940.28 as follows [14]: 7 gm of oil sample was put into a dry and clean 250 ml Erlenmeyer flask, then 50 ml of 96% ethanol was neutralized with 0.1 N aqueous NaOH solution in the presence of 2 ml phenolphthalein solution to produce faint permanent pink, then the neutralized ethanol was added to the oil in the flask, then the mixture was shaken vigorously and boiled on a hot plate for two minutes and then titrated with 0.1 N aqueous NaOH solution until permanent faint pink color appeared and persisted one minute.

#### 2.14.2. Peroxide Value

Peroxide value of olive oil samples was determined by using the AOAC method number 965.33, about 5 g of oil was weighed into 250 ml glass-stoppered conical flask, then 30 ml of acetic acid-chloroform solution (3 : 1 by volume) was added with swirling to dissolve oil completely, then 1 ml of saturated potassium iodide solution was added, then the flask was quickly stoppered and let to stand with occasional shaking for 1 minute in the dark, thereafter, 30 ml of freshly boiled and cooled water were added and flask contents were titrated with 0.01 N sodium thiosulfate solution with vigorous shaking until yellow color had almost gone, and about 0.5 ml of starch solution was added and titration was continued with vigorous shaking to release all iodine from chloroform layer, until the blue color just disappeared. Blank determination is conducted in the same way without the sample (blank is composed of all additions except oil sample).

#### 2.14.3. *K*_232_ and *K*_270_

1% solution of olive oil samples was prepared in cyclohexane (0.25 g oil in 25 ml cyclohexane), and the absorbance of the solution was measured at *λ* of 232 nm and 270 nm for *K*_232_ and *K*_270_, respectively.

### 2.15. Statistical Analysis

Three samples of oil were independently analyzed and all of the determinations were carried out in triplicate. The results are expressed as means ± standard deviations. All statistical analyses were carried out using SAS (SAS Institute Inc., Cary, USA, Release 8.02, 2001). Comparisons of means were carried out using the GLM procedure using one-way analysis of variance (ANOVA). The Bonferroni procedure was employed with multiple *t*-tests in order to maintain an experimentwise of 5%.

## 3. Result and Discussion

### 3.1. Extraction of Polyphenolic Compounds

Upon using the extraction method adopted using ethyl acetate as solvent, polyphenolic extracts were recovered from OMWW with a yield of about 1% (wt/wt). The polyphenols were analyzed using HPLC-PDA after filtration using proper dilution.

### 3.2. HPLC Analysis

**Figure 2:**
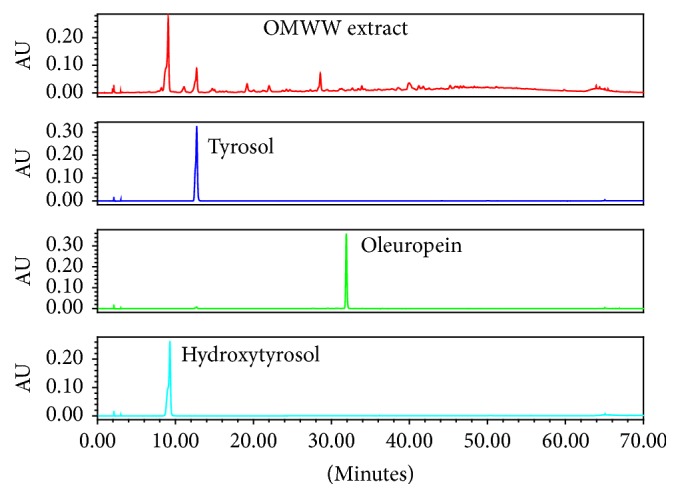
Chromatograms of OMWW ethyl acetate extract, hydroxytyrosol, tyrosol, and oleuropein (100 ppm concentration each).

The quantitation of the diluted samples was achieved based on linear calibration curves. Two solvents were utilized, namely, ethyl acetate and a mixture of ethyl acetate and methanol (2 : 1; v/v).  Figure 2 shows the chromatogram of hydroxytyrosol, tyrosol, and oleuropein with retention times of 9, 12, and 31 minutes, respectively. Results showed that OMWW ethyl acetate extract has only hydroxytyrosol and tyrosol (Figure 2) at a concentration of 371 ± 2.3 mg/L and 272.8 ± 2.1 mg/L, respectively. Oleuropein was not detected in the OMWW ethyl acetate extract; see Figure 2.

Methanol/ethyl acetate (2 : 1 v/v) was used as extraction solvent of OMWW and the amount of hydroxytyrosol and tyrosol was determined based on the linear calibration curves. The amount of tyrosol and hydroxytyrosol in OMWW was found to be 180.51 ± 1.21 and 300.22 ± 2.31 mg/liter, respectively. When comparing methanol/ethyl acetate solvent mixture with ethyl acetate as an extraction solvent for OMWW constituents, it turned out that ethyl acetate alone is superior.

**Figure 3:**
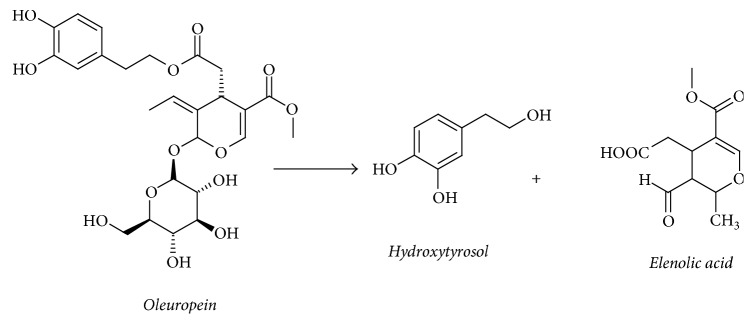
Enzymatic hydrolysis of oleuropein.

The amount of hydroxytyrosol was found to be higher than that of tyrosol in the OMWW, while there was no oleuropein in OMWW extract as it was apparently hydrolyzed enzymatically to hydroxytyrosol and elenolic acid, as shown in Figure 3.

### 3.3. Determination of Total Phenolic Content (TPC)

Total phenolic content of OMWW was determined by Folin-Ciocalteu method, and the results were expressed in g GAE/liter of OMWW using calibration curve at different concentrations of gallic acid. From the calibration curve, the amount of total phenolic content was determined and expressed in grams of gallic acid per one liter of OMWW. Results revealed that TPC of OMWW analyzed in this study is 2500 ± 10 mg gallic acid/liter OMWW.

### 3.4. Total Flavonoid Content (TFC)

TFC was determined by spectrophotometric method at 510 nm and results were expressed in mg of catechin per liter of OMWW using calibration curve at different concentrations of catechin. Results showed that TFC is 499.3 ± 5.1 mg catechin/L of OMWW.

### 3.5. Antioxidant Activity (AA)

Evaluation of antioxidant activity is becoming increasingly relevant in the field of nutrition since it provides useful information with regard to health promoting and functional quality of fruits, vegetables, or medicinal plants. This parameter accounts for the presence of efficient oxygen radical scavengers, such as phenolic compounds. The antioxidant activity of phenolics is mainly due to their redox properties, which make them act as reducing agents, hydrogen donors, and singlet oxygen quenchers [15].

There are two types of antioxidant assays used to evaluate the antioxidant activity of plant extracts. The first category measures the potential of plant extracts to reduce ions or oxidants (to act as reducing agents) like ferric ion and cupric ion. The main two assays of this antioxidant activity category are FRAP (measures the reduction potential of ferric to ferrous ion) and CUPRAC (measures the reduction of cupric to cuprous ion). The second category of antioxidant activity measures the ability of plant extracts to scavenge free radicals. DPPH and ABTS assays (where DPPH and ABTS are stable free radicals) are the two main examples of this category. These assays are used because they are quick and simple to perform, and reaction is reproducible and linearly related to the molar concentration of the antioxidant(s) present.

#### 3.5.1. Reducing Potential of OMWW

*(1) Ferric Reducing Antioxidant Power (FRAP)*. FRAP assay measures the reducing potential of an antioxidant reacting with a ferric tripyridyltriazine (Fe^3+^–TPTZ) complex and producing a colored ferrous tripyridyltriazine (Fe^2+^–TPTZ). The reducing properties are associated with the presence of compounds which exert their action by breaking the free radical chain by donating a hydrogen atom. The reduction of Fe^3+^–TPTZ complex to blue-colored Fe^2+^–TPTZ occurs at low pH.

The antioxidant test based on FRAP assay of OMWW extract was expressed in mM Fe^+2^/g. Calibration curve of different concentrations of Fe^+2^ was prepared and absorption was taken at 593 nm. Result showed that FRAP of OMWW is 2193 ± 15.5 mM Fe^+2^/L OMWW.

*(2) Cupric Reducing Antioxidant Power (CUPRAC)*. Although FRAP antioxidant assay has been very popular among researchers, CUPRAC assay is a relatively new assay developed by Apak et al. [14]. It utilizes the copper(II)-neocuproine [Cu (II)-Nc] reagent as the chromogenic oxidizing agent and is based on the cupric reducing ability of reducing compounds to cuprous. Results were expressed as mg Trolox/L of OMWW. Calibration curve of different concentrations of Trolox was prepared and absorption was taken at 450 nm. Results showed that CUPRAC of OMWW is 433 ± 5.5 mg Trolox/liter of OMWW.

#### 3.5.2. Free Radical Scavenging Ability of Plant Extracts

*(1) DPPH Assay. *DPPH is a free radical compound and has been widely used to test the free radical scavenging ability of various samples [16]. It is a stable free radical with a characteristic absorption at 517 nm that was used to study the radical scavenging effects of extracts. As antioxidants donate protons to this radical, the absorption decreases. Antioxidants, on interaction with DPPH, transfer either an electron or hydrogen atom to DPPH, thus neutralizing its free radical character [17]. The color changed from purple to yellow and the absorbance at wavelength 517 nm decreased.

DPPH assay is based on the ability of the stable free radical 2,2-diphenyl-1-picrylhydrazyl to react with hydrogen donors including phenolics. The bleaching of DPPH solution increases linearly with increasing amount of extract in a given volume. Results were expressed as mg Trolox/liter of OMWW. Calibration curve of different concentrations of Trolox was prepared and absorption was taken at 515 nm. Result showed that DPPH of OMWW is 1406 ± 13.6 mg Trolox/liter of OMWW.

*(2) ABTS Assay*. The free radical scavenging capacity of OMWW extracts was also studied using the ABTS radical cation decolorization assay, which is based on the reduction of ABTS•+ radicals by antioxidants of the OMWW extract tested. Result showed that ABTS of OMWW is 6.3 ± 0.2 mg Trolox/liter of OMWW.

Table 1 summarizes the results of TPC, TFC, and antioxidant activity (FRAP, CUPRAC, ABTS, and DPPH) of OMWW extract.

### 3.6. Antimicrobial Activity

#### 3.6.1. Antibacterial Activity

The effect of OMWW extract was tested on both gram positive and gram negative bacteria and zone of inhibition was measured. Results showed that OMWW extract has an activity on gram positive bacteria* (Staphylococcus aureus)* with a zone of inhibition equal to 23 mm. Also it has an effect on gram negative bacteria* (Escherichia coli)* with a zone of inhibition of 25 mm, compared to that of standard antibiotics which was 10 mm for penicillin used against* S. aureus* and 13 mm for gentamicin used against* E. coli*. Results showed that OMWW extract has higher antibacterial activity against both gram positive and negative bacteria compared to well-known antibiotics.

#### 3.6.2. Antifungal Activity

The effect of OMWW extract was also tested on a fungi and yeast. The OMWW extract was found to be effective against* Candida albicans* yeast that causes Candidiasis (thrush), with zone of inhibition of 23 mm. The OMWW extract was found also effective against* Aspergillus niger*, the filamentous fungus that shows growth of colony diameter about 20% of that for the colony that was grown on the surface of agar without extract (positive control).

### 3.7. OMWW Extract as Preservative

Based on the results of this study which showed that OMWW extract is active against bacteria and fungi, it can be used as preservative in food.

#### 3.7.1. Effect of OMWW Extract on Olive Oil Quality

Olive oil is considered to be relatively affected by storage conditions such as temperature and humidity. Such could be noticed in terms of its acidity and rancidity. Quality of olive oil is evaluated by measuring acid value, peroxide value, *K*_232_, *K*_270_, and total phenolic content.

Free acidity is an important parameter that defines the quality of olive oil and is defined as a percentage as grams of free fatty acids (expressed as oleic acid, the main fatty acid present in olive oil) in 100 grams of oil. As defined by the European Commission Regulation Number 2568/91 and subsequent amendments, the highest quality olive oil (extra-virgin olive oil) must feature a free acidity lower than 0.8%. Virgin olive oil is characterized by acidity between 0.8% and 2%, while lampante olive oil (a low quality oil that is not edible) features a free acidity higher than 2%. The increase of free acidity in olive oil is due to free fatty acids that are released from triglycerides.

Oxidation of vegetable oils during storage modifies their organoleptic properties, affecting the shelf life of this product. The oxidative process depends on illumination, fatty acid composition, availability of oxygen, temperature, and nature and concentration of the antioxidant and prooxidant minor components. However, oil stored in bulk is kept away from light and air, and bottled oil is exposed to light only at the retail outlet [18].

Therefore, the main factors affecting oil shelf life are the minor components, the fatty acid composition of the lipid matrix, and the storage temperature. In most seed oils, tocopherols are the main antioxidants, whereas in virgin olive oils, a fair correlation has been found between total phenols and oxidative stability, measured both at low temperature and at high temperature [10].

Peroxide value is an olive oil quality parameter as acid value. The peroxide value is defined as the amount of peroxide oxygen per 1 kilogram of fat or oil. Traditionally this was expressed in units of milliequivalents or in millimoles per kilogram. The unit of milliequivalent has been commonly abbreviated as mequiv or even as meq. Detection of peroxide gives the initial evidence of rancidity in unsaturated fats and oils. Other methods are available, but peroxide value is the most widely used. It gives a measure of the extent to which an oil sample has undergone primary oxidation. The double bonds found in fats and oils play a role in autoxidation. Oils with a high degree of unsaturation are most susceptible to autoxidation. The best test for autoxidation (oxidative rancidity) is determination of the peroxide value. Peroxides are intermediates in the autoxidation reaction. Autoxidation is a free radical reaction involving oxygen that leads to deterioration of fats and oils which form off-flavors and off-odors. Peroxide value, concentration of peroxide in an oil or fat, is useful for assessing the extent to which spoilage has advanced.

*K*_232_ and *K*_270_ are other two quality indices of olive oil. The UV spectrum involves the absorption of fatty acids, in particular, the 230–270 nm, and shows high absorption when conjugated dienes and trienes formed in the autoxidation process from the hydroperoxides of unsaturated fatty acids and their fragmentation products are present. For this reason, the absorbances measured at 232 nm and 270 nm, namely, *K*_232_ and *K*_270_, provide an official method for olive oil quality control, which is capable of detecting product oxidation and adulteration by means of rectified oils [19, 20] since they can give an indication of the level of oxidation to produce primary and secondary products incurred during production and/or storage [21].

In this study, OMWW extract has been introduced and applied on olive oil which has been divided into three parts: First sample contains 1% OMWW extract, second sample contains 0.5% OMWW extract, and third sample is without extract. Then quality parameters of olive oil samples (acid value, peroxide value, *K*_232_, *K*_270_, and total phenolic content) have been undertaken in the day of preparation (time: zero) and after three months.

*(1) Acid Value*. As shown in Table 2, the acid value of olive oil without OMWW analyzed at time zero was 0.9 ± 0.05 which increased to 1.1 ± 0.04 after three months of storage at room temperature which indicates the increase in free acid with time. However percentage acidity of samples with OMWW added at two concentration levels (0.5% and 1%) did not increase as in the samples without OMWW (acid values are 0.93 ± 0.04 and 0.91 ± 0.05 for olive oil samples with 0.5% and 1% OMWW, respectively).

The oil acidity percentage when compared with the original sample is exposed to a change in the course of time. However the value of such acidity in comparison with the samples to which OMWW extract has been added within three months has increased with time intervals; yet the increase has varied to be limited in the sample of 1% and 0.5% of OMWW extract; nevertheless, the sample to which the normal aforesaid extract has not be added has displayed increase in the acidity values. In other words, the application of the OMWW extract on olive oil would lessen the rate of acidity within the course of time and maintain oil to be restored in good condition.

*(2) Peroxide Value*. As in the acid value, peroxide values were found to be affected by the addition of OMWW extract. While the PV of olive oil without OMWW extract increased from 14.1 ± 0.8 to 18.1 ± 1.0 after three months of storage, the peroxide value of olive oil samples with 0.5% and 1% OMWW did not increase with the same rate as in the oil sample without OMWW extract (see Table 2).

The peroxide value of olive oil with 1%, 0.5%, and 0% OMWW equals 14.2 ± 0.8, 15.2 ± 0.7, and 18.1 ± 1.0, respectively. The peroxide value of olive oil when compared with the original sample is exposed to a change in the course of time. However, the value of such rancidity in comparison with the samples to which OMWW extract has been added within three months has increased with time intervals; yet the increase has varied to be limited in the sample of 1% and 0.5% of OMWW extract; nevertheless, the sample to which the normal aforesaid extract has not been added has displayed increase in the acidity values. In other words, the application of OMWW extract on olive oil would lessen the rate of rancidity within the course of time and maintain oil to be restored in good condition.

*(3)  K*_232_ *and K*_270_. *K*_232_ and *K*_270_ were also affected by the addition of OMWW extract to the olive oil. *K*_232_ and *K*_270_ values of olive oil increase after three months of storage. However the value of such rancidity in comparison with the samples to which OMWW extract has been added within three months has increased with time intervals as shown in Table 2; yet the increase has varied to be limited in the sample of 1% and 0.5% of OMWW extract; nevertheless, the sample to which the normal aforesaid extract has not been added has displayed increase in the acidity values. In other words, the application of the OMWW extract on olive oil would lessen the rate of rancidity within the course of time and maintain oil to be restored in good condition.

*(4) Total Phenolic Content*. As shown in Table 2, TPC has increased in olive oil samples that have been treated with OMWW extracts, because OMWW extract contains high amount of phenolic compounds. However, TPC samples without OMWW decreased after three months (see Table 2) due to oxidation of antioxidants phenolic compounds originally present in olive oil. Table 2 summarizes the results of AV, PV, *K*_232_, *K*_270_, and TPC of olive oil with and without OMWW extract.

## 4. Conclusions

Ethyl acetate is found to be an excellent solvent for phenolic compound extraction and enrichment from OMWW. OMWW contains mainly hydroxytyrosol and tyrosol but not oleuropein as the HPLC-PDA results revealed. The phenolic fraction of OMWW gives its high antioxidants activities as reflected by both free radical scavenging ability of OMWW (DPPH, ABTs) and its reducing ability (FRAP, CUPRAC tests). OMWW showed an antibacterial activity against both gram positive and gram negative bacteria, and it also proved to have a significant antifungal activity. The current investigation disclosed many benefits for OMWW including antioxidants for olive oil due to its phenolic content which act as natural antioxidant. Further, it has the potential to act as natural antioxidants and preservatives, thus obviating the environmental problems and making use of it medically and nutritionally. Therefore, oil can be saved by OMWW extract and also increase the content of phenolic compounds which implies increasing antioxidants in it.

## Figures and Tables

**Table 1:** TPC, TFC, and AA (FRAP, CUPRAC, ABTS, and DPPH) of OMWW investigated in this study.

TPC (g gallic acid/L)	TFC (mg catechin/L)	FRAP (mmol Fe^2+^/L)	CUPRAC (mg Trolox/L)	ABTS (mg Trolox/L)	DPPH (mg Trolox/L)
2500 ± 10	499.3 ± 5.1	2193 ± 15.5	433 ± 5.5	6.3 ± 0.2	1406 ± 13.6
RSD = 4.0%	RSD = 1.0%	RSD = 0.7%	RSD = 1.3%	RSD = 3.1%	RSD = 1.0%

**Table 2:** Different quality parameters of olive oil with different concentrations of OMWW extract.

Concentration of OMWW extract in olive oil		Acid value	Peroxide value (milliequivalents O_2 _kg^−1^ oil)	*K*_232_	*K*_270_	TPC (mgGA/g oil)
0.0%	Time zero	0.90^c^ ± 0.05	14.1^c^ ± 0.8	3.44^c^ ± 0.05	0.42^c^ ± 0.03	250.7^c^ ± 2.1
	Time after three months	1.1^a^ ± 0.04	18.1^a^ ± 1.0	3.64^a^ ± 0.04	0.49^a^ ± 0.04	241.2^d^ ± 1.6
0.5%		0.93^b^ ± 0.04	15.2^b^ ± 0.7	3.48^b^ ± 0.06	0.44^b^ ± 0.03	260.3^b^ ± 1.1
1.0%		0.91^c^ ± 0.05	14.2^c^ ± 0.8	3.46^c^ ± 0.05	0.43^c^ ± 0.04	267.2^a^ ± 1.6

*Note*. Results are expressed as average of three samples. Different small letters within column indicate significant difference (*p* < 0.05, *n* = 3).
